# Identification of transcription factors of nitrate reductase gene promoters and NRE2 *cis*-element through yeast one-hybrid screening in *Nicotiana tabacum*

**DOI:** 10.1186/s12870-019-1724-z

**Published:** 2019-04-16

**Authors:** Huijuan Yang, Yan Zhou, Yuning Zhang, Jing Wang, Hongzhi Shi

**Affiliations:** College of Tobacco Science, Henan Agricultural University, No. 95 Wenhua Road, Zhengzhou, 450002 Henan China

**Keywords:** Nitrate reductase, Transcriptional factor, Yeast one-hybrid library, *cis*-element

## Abstract

**Background:**

This study aimed to identify the transcription factors of nitrate reductase genes (*NIA1* and *NIA2*) promoters and hypothetical *cis*-element of NRE2. Based on the constructed cDNA library of *Nicotiana tabacum* K326, a yeast one-hybrid system was established using the Matchmaker® Gold Yeast One-Hybrid Library Screening System from Clontech. The transcription factors of *NIA1* and*NIA2* promoters and NRE2 *cis*-elements were screened.

**Results:**

After sequencing and bioinformatics analysis, 15 cDNA sequences were identified: 9 for *NIA1* (including XP_016503563.1 and NP_001312236.1), 3 for *NIA2* (including XP_016510250.1), and 3 for *NRE2* (including XM_016576899.1). XP_016503563.1 was annotated in PREDICTED: CRM-domain containing factor CFM3, and NP_001312236.1chloroplastic/mitochondrial-like in *Nicotiana tabacum*. NP_001312236.1 was annotated in Sulfite oxidase-like of *Nicotiana tabacum*. XP_016510250.1 was annotated as PREDICTED: uncharacterized protein LOC107827596 in *Nicotiana tabacum*. XM_016576899.1 was annotated in PREDICTED: *Nicotiana tabacum* RING-H2 finger protein ATL16-like (LOC107759033).

**Conclusion:**

A yeast one-hybrid library was successfully constructed. The identified transcription factors may provide a theoretical basis for the study of plant nitrate reductase.

## Background

Nitrogen is a fundamental constituent of cell components such as nucleic acids, amino acids, proteins, membranes, and cell walls. Additionally, nitrogen is quantitatively the most important nutrient that plants acquire from soil [1]. Nitrogen deficiency exerts severe pressure on plant growth, development, and photosynthesis, ultimately limiting plant productivity [2–4]. Nitrate is a primary nitrogen source for most plants [5]. Furthermore, it functions as a signal molecule that promptly triggers changes in metabolism, growth, and gene expression in plants [6, 7]. The genes encoding the enzymes of nitrate reductase (NR) and nitrite reductase (NIR) are typical nitrate-inducible genes [8]. Overexpression of NR and NIR genes has an influence on the nitrogen metabolism of plants. Davenport et al. [9] reported that overexpression of the NIR gene in tobacco could affect both nitrogen and carbon metabolism, extend stay-green time, elevate NR activity, reduce nitrate, proline, glutamine, and glutamate contents.

Plant growth, development, and their responses to the environment are regulated by the differential expression of genes, of which the major mechanism is transcriptional regulation controlled by transcription factor (TF) which binds to DNA *cis*-elements located in the gene promoters [10]. Currently, several identified TFs and other factors that regulate transcription are involved in nitrate signal transduction, such as MADS-box TF *Arabidopsis* nitrate-regulated 1 (ANR1) [11], NIN-like protein 7 (NLP7) [12], and the lateral organ boundary domain (*LBD*) gene family transcription factors of LBD37, LBD38, and LBD39 [13]. Furthermore, previous studies have shown the presence of a conserved sequence (GACCCTT-N (9–10)-AAG), a nitrate-responsive *cis*-element (NRE), located in the NIR gene promoter in a variety of plants that is necessary for gene expression regulation in the nitrate-mediated signal transduction pathway [14, 15]. Nevertheless, until now, the TF acting on the NR gene promoter and NRE that responds to a nitrate signal in the promoter remains unclear.

According to the existing NRE sequence, we found that there were similar but slightly different sequences (GACCCTA-N (10)-AAG) in the NR genes (*NIA1* and *NIA2*) promoters of *Nicotiana tabacum*, which was tentatively named as NRE2. Our present study aimed to investigate the role of NRE2 in *NIA1* and *NIA2* promoters and to identify the TFs specifically binding to *NIA1*, *NIA2*, and NRE2 based on a yeast one-hybrid system. The findings of this study may not only help to further understand the regulatory pathway of nitrate signal response in tobacco but also provide new ideas for future research into nitrate signal transduction in other plants.

## Methods

### Cloning of NIA1and NIA2 promoters

We searched the ATG upstream sequences of *NIA1*and *NIA2*genes in the National Center of Biotechnology Information (NCBI) database, and a 937 bp sequence was intercepted from each gene for the design of primers. The primers were as follows: NIA1 (forward), TATATATATGACCCTGCAATGAAAG; NIA1 (reverse), AGATTATTCTAAAAAAGAAAGAGAGAT; NIA2 (forward), TACATACAAGGGCGCGAATAA; and NIA2 (reverse), AGATTATTCTAAAAAAAGAATATGAATG. Using the tobacco genome as template, polymerase chain reaction (PCR) amplification was performed to generate fragments which were then sequenced and compared with the target fragments.

### Construction of the β-glucuronidase (GUS) expression vector

The tobacco cultivar “Nicotiana benthamiana” was from the national tobacco cultivation physiological and biochemical base, Agricultural University Of He’nan, China. And *Agrobacterium* (EHA105; Shanghai Zeye biotechnology co., LTD, China) was used for was used for the transformation experiments. The possible NO_3_^−^ signal response element (NRE2) sequence (GACCCTACGGGCGTAAAAAG) was predicted from PlantCARE (http://bioinformatics.psb.ugent.be/webtools/plantcare/html/), a database of plant *cis*-acting regulatory elements, enhancers, and repressors [16]. After the analysis of promoter sequences of NIA1 and NIA2 genes, we found NRE2 is located at -281 bp ~ − 262 bp upstream of NIA1 gene and -332 bp ~ − 313 bp upstream of NIA2 gene. Next, the *NIA1* and *NIA2* promoters were modified by the knockout of NRE2 element, and inserting four tandem NRE2 elements. The primers used for amplification of modified promoters were the same as the primers described above. The 35S promoter of vector pCAMBIA-NPT-GUS was digested with EcoR I and Hind III. The original and modified *NIA1* and *NIA2* promoters were digested with EcoR I and Hind III, respectively, and inserted in place of the 35S sequence in pCAMBIA-NPT-GUS using T4 DNA ligase. The ligated products were then transformed into *Escherichia coli* DH5α competent cells and recombinant clones were identified using colony PCR. Recombinant expression vectors that expressed GUS genes [pCAMBIA-NPT-NIA1-P-GUS, pCAMBIA-NPT-NIA1-P-△NRE2-GUS (meaning without the NRE2 element), pCAMBIA-NPT-NIA1-P-(4 × NRE2)-GUS (meaning with four tandem NRE2 elements), pCAMBIA-NPT-NIA2-P-GUS, pCAMBIA-NPT-NIA2-P-△NRE2-GUS, and pCAMBIA-NPT-NIA2-P-(4 × NRE2)-GUS] were selected. The recombinant plasmids were validated by PCR.

### Agrobacterium-mediated transient transfection assay

*Nicotiana benthamiana* (grown for 45 days) was used in this experiment. *Agrobacterium* EHA105 was grown overnight in 5 mL YEB medium supplemented with kanamycin (50 μg/mL), rifampicin (25 μg/mL), and streptomycin (50 μg/mL). Next, 1.5 mL bacterial solution was transferred into a sterile tube and centrifuged for 10 min at 1000×*g*. After washing, the bacterial solution was diluted 10-fold to obtain an optical density (OD) of 0.3–0.4 at 600 nm. The tobacco plant was placed under a white fluorescent lamp for 1 h to ensure that the stomata were fully opened. Two large leaves were selected and the cuticle was removed through friction (about 0.5 cm^2^). The *Agrobacterium* solution was injected into the area lacking a cuticle using a 1-mL syringe. After 48 h of transfection, the leaf tissue was collected and assayed immediately.

### Histochemical GUS staining and quantitative measurements

GUS reporter gene enzymatic activity was determined using histochemical staining. Briefly, the mature leaves of transformants were collected and cut into small round pieces using a punch. These small round pieces were stained with GUS staining solution for 6 h at 37 °C, followed by elution with 50, 70, and 100% ethanol until completely decolorized. Additionally, GUS activity was quantitative analyzed using a fluorometric assay, with 4-umbelliferyl-D-glucuronide as substrate according to a previous study [17]. Protein content was determined via bradford protein assay [18].

### Acquisition of bait sequence

The upstream promoter sequences (about 1 kb) of *NIA1* and *NIA2* were cloned based on primers. To improve the screening efficiency, four NRE2 sequences were tandemly iterated. After ligating Xho I and Hind III sites that were the same in all vector plasmids to both ends of the sequences above, bait sequences with four tandem NRE2 elements (4 × NRE2) were obtained and named “4 box.”

### Construction of a tobacco cDNA library

Tobacco (K326), provided by the tobacco cultivation and physiology laboratory of Henan Agricultural University, was used in this experiment. A cDNA library of K326 was constructed via a switching mechanism at the 5′ end of RNA transcript (SMART™). Briefly, the root, stem, leaf, and flower of K326 at different developmental stages were quickly frozen in liquid nitrogen and then stored at − 70 °C. Total RNA from different tissues was extracted and mixed together. After purification of total RNA using NucleoSpin RNA II (740,955.20; Clontech, Palo Alto, CA, USA), poly A+ mRNA was enriched using a NucleoTrap mRNA Kit (740,655; Clontech). The first strand of cDNA was then synthesized based on the SMART technique and duplex-specific nuclease (DSN) according to the Matchmaker® Gold Yeast One-Hybrid Library Screening System (Clontech). Double-stranded cDNA was synthesized through long-distance (LD)-PCR. Following purification using a chromatographic column, a normalized cDNA library of K326 was obtained.

### Construction of a recombinant bait plasmid

The *NIA1* and *NIA2* promoter sequences, 4 box oligonucleotides, and the pAbAi vector were digested with XhoI and HindIII, respectively. The enzyme-digested products were purified on agarose gel and ligated into the linearized bait sequences and pAbAi vector (carrying the URA3and AUR1-C genes) using T4 ligase. The ligated products were transformed into *E. coli* DH5α competent cells and positive recombinant clones were screened using Luria-Bertani solid media containing ampicillin. The plasmids were then extracted and digested with Xho I and Hind III before PCR electrophoresis to confirm the insertion of the target fragment. The recombinant plasmids were identified by DNA sequencing.

### Transformation of linearized bait plasmids into yeast cells

Yeast competent cells were prepared using the LiAc method. *Bbs*B I-linearized recombinant bait plasmids (pAbAi-P: NIA1, pAbAi-P: NIA2, and pAbAi-P: (4 × NRE2)) (1 μg) were integrated into the genome of yeast (Y1HGold). The transformed competent cells were then transferred onto solid agar synthetic defined (SD) medium-Ura and incubated for 3 days. Single clones were then identified via colony PCR using Matchmaker Insert Check PCR Mix 1 (Clontech). The expected size of PCR products was 1.35 kb.

### Determination of basal expression of the reporter gene

Healthy yeast colonies grown in SD medium-Ura were selected and resuspended in 0.9% NaCl. After adjusting to obtain OD_600nm_ values of approximately 0.002, yeast strain was transferred onto each of the following solid media: SD-Ura without aureobasidin A (AbA), SD-Ura with 100 ng/mL AbA, SD-Ura with 200 ng/mL AbA, SD-Ura with 300 ng/mL AbA, SD-Ura with 400 ng/mL AbA, SD-Ura with 500 ng/mL AbA, SD-Ura with 600 ng/mL AbA, SD-Ura with 700 ng/mL AbA, SD-Ura with 800 ng/mL AbA, SD-Ura with 900 ng/mL AbA, and SD-Ura with 1000 ng/mL AbA. The colonies were allowed to grow for 2–3 days at 30 °C. The minimum AbA concentration that completely inhibited colony growth was determined and used for further library screening.

### Construction and screening of a yeast one-hybrid library

Purified double-stranded cDNA (5 μg) and SmaI-linearized pGADT7-Recexpression vector (3 μg) were mixed to co-transform bait yeast using the Yeast maker Yeast Transformation System 2 (Clontech). Yeast cell suspensions were diluted to 1/10, 1/100, 1/1000, and 1/10000, and 100-μL diluents were coated onto SD-Leu plates to screen the total number of colonies. The remaining yeast cell suspensions (approximately 15 mL) were coated on corresponding SD-Leu/AbA plates (200 μL per plate) to preliminarily screen the positive colonies. The number of colonies on SD-Leu was calculated 3–5 days later using the following formula: total number of colonies = colony-forming unit/volume of coated plate × dilution rate × total volume of cell suspension.

### Confirmation of positive interaction and plasmid extraction

Healthy single clones from the SD-Leu/AbA plate were transferred into new SD-Leu medium containing AbA and incubated at 30 °C for 3–5 days. The screened yeast single clones were then transferred onto SD-Leu/AbA for repeated screening. The colonies that eventually grew on SD-Leu/AbA plates were selected for yeast colony PCR using Matchmaker Insert Check PCR Mix 2 (Clontech) to obtain the inserted fragment of cDNA library. A small amount of the product was identified by 1% tris-acetate-ethylenediamine tetraacetic acid (TAE) agarose/ethidium bromide (EtBr) gel electrophoresis. After purification, the PCR products were sequenced at the Beijing Genomics Institute, Shenzhen, China, with T7 as primer. Bioinformatic analyses were then performed on the data to analyze the cDNA library quality and identify positive clones. Sequences with biological significance were selected and the corresponding plasmids were extracted using an Easy Yeast Plasmid Isolation Kit (630,467; Clontech) and stored at − 20 °C.

### Statistical analysis

All data processing was done using SPSS 21.0 software, and the measurement data have been expressed in mean ± SD. Statistical differences between two groups were tested by Student’s t-test., and a *P* < 0.05 indicates statistical significance.

## Results

### Cloning of NIA1 and NIA2 promoters

**Fig. 1:**
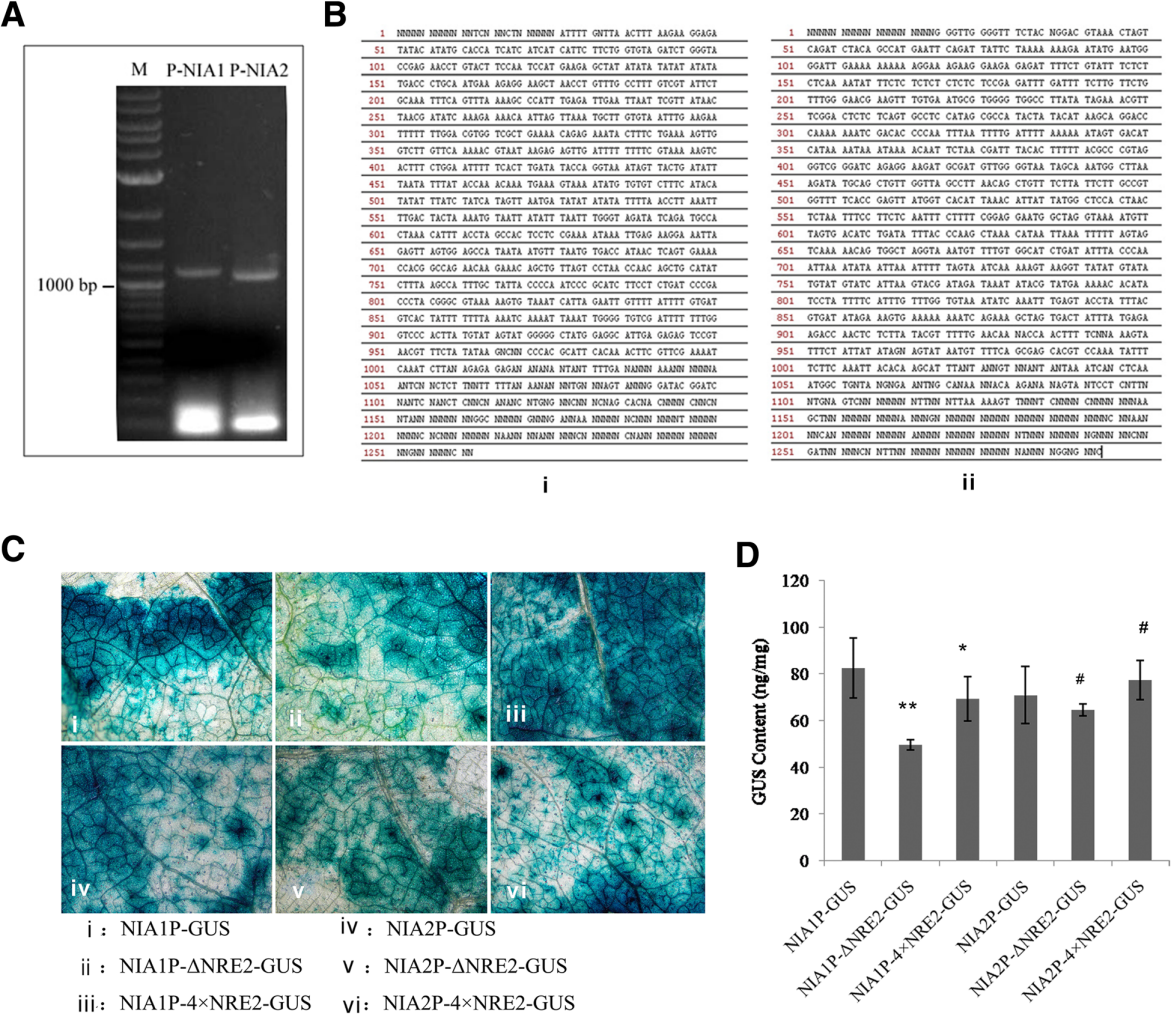
Cloning of *NIA1* and *NIA2* promoters and GUS expression levels. Electrophoretic diagram of cloned *NIA1* promoter and *NIA2* promoter (**a**). M: DNA marker; P-NIA1: the amplified fragment of *NIA1* gene promoter; P-NIA2: the amplified fragment of the *NIA2* gene promoter. Sequencing results of cloned (i) *NIA1* promoter and (ii) *NIA2* promoter (**b**). GUS histochemical staining results of tobacco leaves transformed by different promoter sequences (**c**) and the GUS content of tobacco leaves transformed by different promoter sequences measured by quantitative assay (**d**). * represents *P* < 0.05, and ** represents *P* < 0.01 when compared with normal *NIA1* promoters; # represents *P* < 0.05 when compared with normal *NIA2* promoters

The tobacco *NIA1* and *NIA2* gene promoter sequences were cloned based on the designed primers. The amplified products were analyzed by agarose gel electrophoresis, and their lengths were consistent with target lengths (Fig. 1a). Sequencing results, as shown in Fig. 1b, suggest that *NIA1* and *NIA2* promoter sequences were successfully cloned.

### GUS activity assay

In order verify whether the change of NRE2 elements could influence the expression of NIA1 and NIA2 genes, we added four NRE2 in NIA1 and NIA2 promoters ((pCAMBIA-NPT-NIA1-P-(4 × NRE2)-GUS and pCAMBIA-NPT-NIA2-P-(4 × NRE2)-GUS)), which will lead to the improvement of the screening efficiency. Moreover, the GUS expression can be directly reflected by staining. As shown in Fig. 3c, the results of GUS staining showed that, when compared with the control (staining results of the leaves transformed by normal *NIA1* and *NIA2* promoters), GUS expression levels significantly decreased after knockout of the NRE2 elements in *NIA1* and *NIA2* promoters. Additionally, GUS expression levels in the leaves transformed by *NIA1* and *NIA2* promoters containing 4 tandem NRE2 elements were significantly higher than that in the leaves transformed by *NIA1* and *NIA2* promoters lacking NRE2 sequences. Moreover, a quantitative assay also revealed that knockout of the NRE2 sequences in the *NIA1* and *NIA2* promoters could significantly reducethe GUS expression levels compared to those of the control, respectively (both *P* < 0.05; Fig. 1d). Moreover, the leaves transformed by *NIA2* promoter containing 4 tandem NRE2 elements showed higher GUS expression (more than 10%) than leaves in the control group (both, *P* < 0.05).

### Identification of bait yeast strain and determination of AbA basal expression

**Fig. 2:**
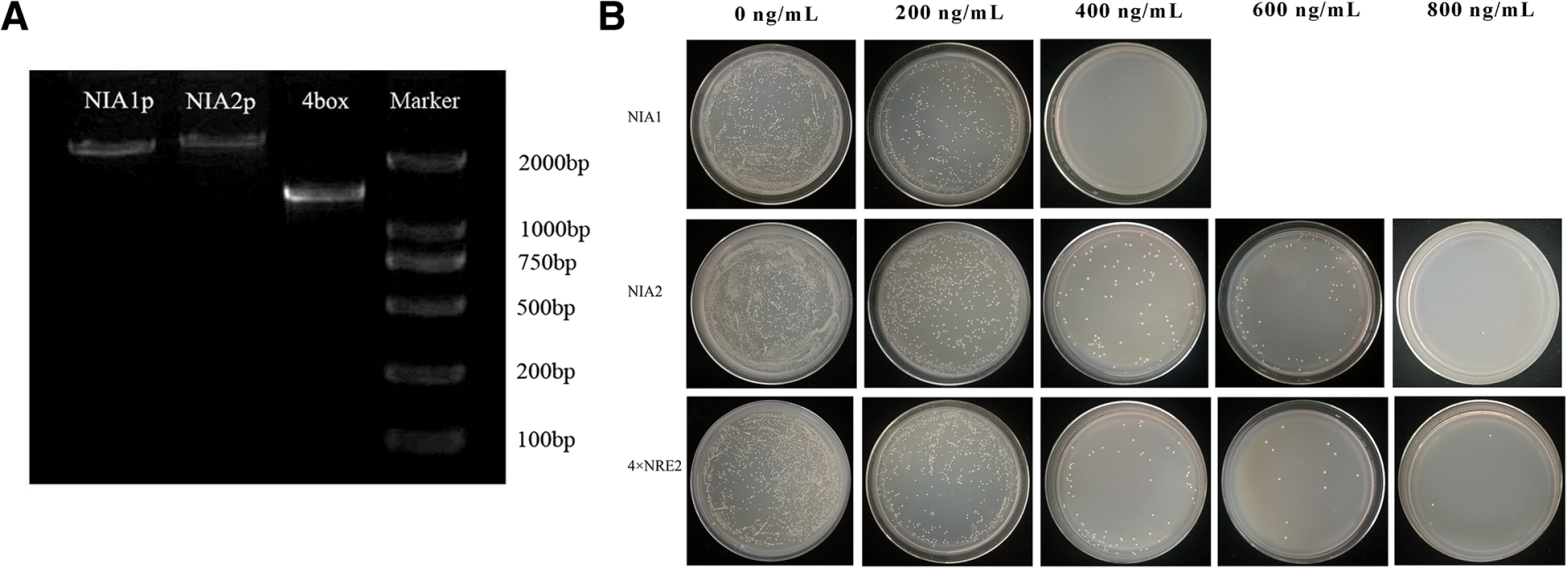
Identification of bait-yeast strains by yeast colony PCR (**a**). NIA1p: PCR product of colony transformed by pAbAi-NIA1p plasmid; NIA2p: PCR product of colony transformed by pAbAi-NIA2p plasmid; 4box: PCR product of colony transformed by pAbAi-(4 × NRE2) plasmid; Marker: DL2000 DNA marker. Identification of the minimum inhibitory concentration of AbA (**b**)

Three bait recombinant vectors (pAbAi-P: NIA1, pAbAi-P:NIA2, and pAbAi-P: (4 × NRE2)) were transformed into Y1HGold. Colony PCR showed that the obtained fragment length was consistent with the expected size of PCR products (1.35 kb not including the inserted fragment, Fig. 2a), suggesting that the three bait recombinant vectors were successfully transformed into yeast cells.

To omit the influence of target sequence recognition by endogenous yeast transcription factors, the minimum inhibitory concentration of AbA (basal expression level of reporter gene) was measured. As shown in Fig. 2b, the minimum concentrations of AbA needed to suppress basal expression of the *NIA1*, *NIA2*, and 4box bait strains were 400 ng/mL, 800 ng/mL, and 800 ng/mL, respectively.

### Construction of a K326 cDNA library

**Fig. 3:**
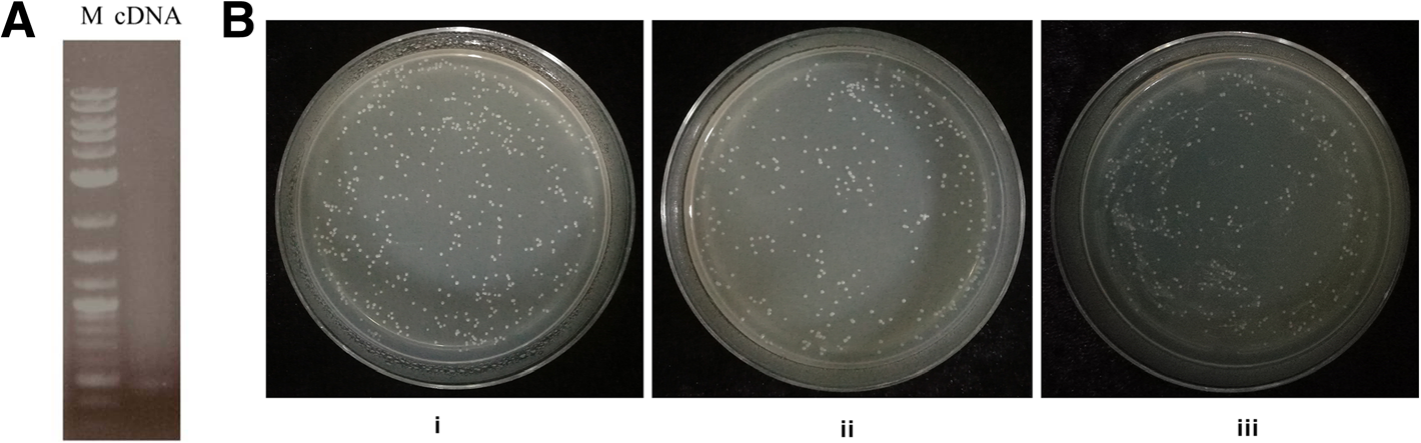
Electrophoretic diagram of cDNA library (**a**). M: DNA marker; cDNA: electrophoretic feature of constructed cDNA library. Colony map of calculating library capacity (**b**). i, Growth of 1/100 dilution of NIA1P transformant; ii, Growth of 1/100 dilution of NIA2P transformant; iii: Growth of 1/100 dilution of 4 × NRE2 transformant

Electrophoretic identification of the constructed K326 cDNA library showed that the bands were dispersive and evenly distributed (Fig. 3a), indicating that the expression of each gene in the library was average. This result suggests that the K326 normalized cDNA library was successfully constructed.

### Screening of a yeast one-hybrid library and extraction of yeast plasmids

NIA1P, NIA2P, and 4box yeast cell suspensions were diluted to 1/100, and 304, 264, and 240 colonies, respectively, were obtained on SD-Leu plates (Fig. 3b). The total number of colonies was 4.6 × 10^6^, 3.96 × 10^6^, and 3.6 × 10^6^, respectively. Colony PCR and electrophoretic identification showed that the insert fragment was 200 bp to 2000 bp in length, indicating that the capacity and quality of the constructed library was adequate.

The identification analysis with PCR assay was performed on a total of 310 positive colonies with *NIA1* promotor sequences, and 97 PCR products with bright bands and long fragments were sequenced and analyzed via bioinformatics. Finally, 9 sequences of *Nicotiana* were selected, including 7 predicted proteins (such as XP_016503563.1), 1 hypothetical protein, and 1 sulfite oxidase-like protein (NP_001312236.1, Table 1). Meanwhile, the identification analysis with PCR assay was performed on 302 positive colonies with *NIA2* promoter sequences, of which 128 PCR products were sequenced and analyzed, and 3 sequences with biological significance were selected. The 3 sequences were from *Populus* (XP_006380094.1), *Lupinus* (CP023132.1), and *Nicotiana* (XP_016510250.1), respectively (Table 2). Moreover, 3 sequences were selected from 288 positive colonies with 4box sequences, which originate from *Gossypium* (XM_017753776.1), *Ustilaginoidea* (KDB15619.1), and *Nicotiana* (XM_016576899.1, Table 3).

**Table 1:** Screening results of *NIA1* promotor transcriptional factors

Number	Fragment length (bp)	Blastx/Blastn	NCBI number	Biological annotation	Species
51a	449/269	blastx	XP_009771766.1	PREDICTED: uncharacterized protein At2g39795, mitochondrial-like	*Nicotiana sylvestris*
66	333/129	blastn	XR_001647849.1	PREDICTED: *Nicotiana tabacum* uncharacterized LOC107785360 (LOC107785360), ncRNA	*Nicotiana tabacum*
68	329/127	blastx	XP_009790808.1	PREDICTED: uncharacterized protein LOC104238210	*Nicotiana sylvestris*
71	314/95	blastx	XP_016503563.1	PREDICTED: CRM-domain containing factor CFM3, chloroplastic/mitochondrial-like	*Nicotiana tabacum*
94	317/115	blastx	YP_173415.1	Hypothetical protein; NitaMp073	*Nicotiana tabacum*
95	377/185	blastn	XM_016598923.1	PREDICTED: CMSS1-like (LOC107778635), transcript variant X2, mRNA	*Nicotiana tabacum*
126	1149/591	blastx	NP_001312236.1	Sulfite oxidase-like	*Nicotiana tabacum*
135	437/236	blastx	XP_016455866.1	PREDICTED: uncharacterized protein LOC107779879	*Nicotiana tabacum*
153	403/196	blastn	XM_016643438.1	PREDICTED: uncharacterized LOC107817585 (LOC107817585), mRNA	*Nicotiana tabacum*

**Table 2:** Screening results of *NIA2* promotor transcriptional factors

Number	Fragment length (bp)	Blastx/Blastn	NCBI number	Biological annotation	Species
7	272/66	blastx	XP_006380094.1	Hypothetical protein POPTR_0008s21830g	*Populus trichocarpa*
25	249/41	blastn	CP023132.1	*Lupinus angustifolius* cultivar Tanjil chromosome LG-20	*Lupinus angustifolius*
72	412/62	blastx	XP_016510250.1	PREDICTED: uncharacterized protein LOC107827596	*Nicotiana tabacum*

**Table 3:** Screening results of 4 × NRE2 transcriptional factors

Number	Fragment length (bp)	Blastx/Blastn	NCBI number	Biological annotation	Species
34	247/42	blastn	XM_017753776.1	PREDICTED: *Gossypium arboreum* uncharacterized LOC108455177 (LOC108455177), mRNA	*Gossypium arboreum*
46	321/130	blastx	KDB15619.1	Glycoside hydrolase family 16	*Ustilaginoidea virens*
48	242/37	blastn	XM_016576899.1	PREDICTED: *Nicotiana tabacum* RING-H2 finger protein ATL16-like (LOC107759033), mRNA	*Nicotiana tabacum*

Furthermore, the target plasmids were extracted and validated via electrophoresis. The inserted fragment was 200 bp to 2000 bp in length.

## Discussion

Yeast one-hybrid screening is widely recognized as a useful technique for the detection of physical interactions between DNA and DNA-binding proteins, such as TFs [19]. The present study successfully constructed yeast one-hybrid libraries of NR gene (*NIA1* and *NIA2*) promoters and NRE2 *cis*-element; moreover, several potential TF genes, such as XP_016503563.1, NP_001312236.1, XP_016510250.1, and XM_016576899.1 were screened.

XP_016503563.1 was identified from the *NIA1* promoter and annotated in predicted: CRM-domain containing factor CFM3, chloroplastic/mitochondrial-like of *Nicotiana*, having homology with predicted protein CFM3 in *Nicotiana tabacum*. The CRM domain is an RNA-binding domain identified in three group II intron splicing factors in chloroplasts, in a family of uncharacterized proteins in plants [20]. Additionally, CRM domain proteins are implicated in the activities of three classes of catalytic RNA, and could interact with catalytic RNA [20]. An in vitro experiment also revealed that the isolated CRM domain in maize has RNA-binding activity [21]. Interestingly, RNA-binding proteins play a key role in the post-transcriptional regulation of gene expression. Besides, some RNA-binding proteins with specific structures can bind to DNA as TFs [22]. CFM3 is a CRM-domain protein related to chloroplast splicing factor CRS1, which dually functions in chloroplast group II intron splicing and mitochondrial gene expression [23]. Therefore, we speculated that XP_016503563.1 may be involved in the transcriptional regulation of the *NIA1* promoter in *Nicotiana tabacum*.

Another gene identified from the *NIA1* promoter, NP_001312236.1, was annotated as a sulfite oxidase-like protein in *Nicotiana tabacum*. Sulfite oxidase is the smallest eukaryotic molybdenum enzyme that utilizes a molybdopterincofactor and aheme group [24]. A study reported that sulfite oxidase and NR have considerable homology, suggesting that they may come from a common family [25]. Notably, Nakamura et al. [26] found that all sulfite oxidases possess a peroxisomal targeting sequence at the C-terminus, and that the peroxisomal targeting sequence could direct polypeptides into peroxisomes in plants, animals, and yeasts. Therefore, the role of sulfite oxidase in transcriptional expression of the NR gene needs to be further verified.

From the *NIA2* promoter, 3 TF genes were identified, among which XP_016510250.1 was annotated in PREDICTED: uncharacterized protein LOC107827596 in *Nicotiana tabacum*. In the Conserved Domain Database, this protein was found to have a pepsin-like aspartic protease domain. Eukaryotic pepsin-like proteases have two domains with similar topological characteristics: C- and N-terminal domains. However, they have limited sequence homology except for the sequences near the active site, indicating that this enzyme may have evolved from ancient copying activity. The active site motif (Asp-Thr/Ser-Gly-Ser) is conserved between retroviruses and eukaryotic proteases, as well as eukaryotic N- and C-terminal pepsin-like proteins. Currently, its relationship with *NIA2* promoter gene expression remains to be explored.

XM_016576899.1 was identified from 4 tandem NRE2 *cis*-elements, which encoded a protein containing RING finger motifs [27]. RING finger proteins contain zinc finger domains consisting of one or several Cys and His residues. Zinc fingers can serve as DNA-binding domains to bind DNA, meeting the structural requirements of TFs. On the basis of the spacing between metal ligands or substitutions at one or more of the metal ligand positions, total 9 RING types were identified: RING-HC, RING-H2, RING-C2, RING-G, RING-mH2, RING-v, RING-D, RING-S/T, and RING-mHC [28]. It was reported that RING finger protein SEVEN IN ABSENTIA OF ARABIDOPSIS2 (SINAT2) may interact with TF RAP2.2 to co-regulate carotenogenesis in *Arabidopsis* leaves [29]. A recent study revealed that NtRHF1 containing a RING motif in its C-terminal region participated in drought stress response via transcriptional regulation of the downstream stress-responsive genes NtCDPK2, NtLEA5, NtAREB, and NtERD10C in tobacco [30]. Therefore, these results indicate that the RING finger family may regulate the transcriptional expression of genes independently or with other transcription factors.

## Conclusions

In conclusion, the identified TF genes from *NIA1* and *NIA2* promoters and the NRE2 *cis*-element (such as XP_016503563.1, NP_001312236.1, XP_016510250.1, and XM_016576899.1) may help us to understand the regulatory pathway of nitrate signal response in *Nicotiana tabacum*. Our results may provide a theoretical basis and possible research directions for investigation of the TFs of the NR gene promoter. Further studies are needed to characterize their functions.

## Abbreviations

ANR1: *Arabidopsis* nitrate-regulated1
DSN: Duplex-specific nuclease
*LBD*: Lateral organ boundary domain
LD: Long-distance
NIR: Nitrite reductase
NLP7: NIN-like protein 7
NR: Nitrate reductase
NRE: Nitrate-responsive *cis*-element
OD: Optical density
PCR: Polymerase chain reaction
TF: Transcription factor
